# The Promise of CRISPR for Human Germline Editing and the Perils of “Playing God”

**DOI:** 10.1089/crispr.2019.0033

**Published:** 2020-02-17

**Authors:** Larry G. Locke

**Affiliations:** University of Mary Hardin-Baylor, Belton, Texas.

## Abstract

In the midst of the media and professional exuberance regarding the potential benefits of CRISPR technology, voices of criticism and caution have also arisen. One of the thorniest such cautions has been the common objection that CRISPR allows bioscientists to “play God,” particularly when it comes to potentially editing the human germline. Many in the biotechnology field are unsure how to address this concern. What does it mean, particularly for bioscientists who may not have any rational or rhetorical categories for God? In this article, I explore possible meanings of “playing God” and the arguments for how those meanings might be applied in the utilization of CRISPR technology for human germline editing. I then test the validity of those arguments and explore potential counterarguments. Finally, I discuss how members of the bioscience community might respond to the objection of “playing God” and contribute to that dialogue in ways that could impact the future of CRISPR development and applications.

## Introduction

Unless you were around to witness the development of immunology by Louis Pasteur in the 1870s, it is hard to imagine a biotechnology that has generated more acclamation than CRISPR. CRISPR technology has been lauded as “the most versatile genomic engineering tool created in the history of molecular biology to date,”^1^ for its “unprecedented potential to revolutionize innovation in basic science,”^2^ and its “huge potential to allow us to cure disease.”^3^ Each of these accolades appeared in academic journals, normally written with a more professionally reserved tone. The popular press has been equally effusive: CRISPR is “taking the scientific world by storm”^4^ and carries “the possibility for inherited diseases to be wiped out entirely.”^5^

The use of CRISPR technology holds significant promise for many clinical applications. Several research trials have already been run using CRISPR to investigate treatments for human immunodeficiency virus type 1, sickle cell anemia, human papilloma virus, and several forms of cancer.^1^ CRISPR has been used to model disease mutations in different animal species.^6^ The comparative affordability, efficiency, scalability, precision, and programmability of CRISPR technology have contributed to its rapid adoption and its eclipse of earlier gene-editing technologies.^7^

Despite its potential, not all of the voices surrounding the development of CRISPR technology have been positive. On the subject of deploying CRISPR to edit the human germline, more cautionary messages appear. This particular application of CRISPR technology has been labeled “an enormous threat”^8^ and “unnatural.”^3^ In *The CRISPR Journal*, Brokowski compared 61 ethics statements produced by various governments, societies, and other organizations.^2^ More than half of these statements held heritable human genome editing (HGE) impermissible, while another 11% held it impermissible at present but were open to its reconsideration in the future.^2^ Those expressing apprehension included the U.S. National Academy of Sciences, Engineering, and Medicine (NASEM)^2^ and the Council of Europe's Committee on Bioethics.^9^

Apprehension regarding the use of CRISPR for HGE derives from several concerns. In 2018, Dijke *et al.* published a meta study of 160 articles on germline modification and identified 79 reasons cited against such applications.^10^ Most identified concerns resounded in terms of safety risks for the child due to on- or off-target effects of the gene editing itself.^10^ Because of the heritability of germline modifications, those concerns included safety risks for subsequent generations.

These concerns are potentially addressable through careful experimentation and ethical research. But an important category of concerns in the Dijke *et al.* study might prove much harder for the bioscience community to address. Of the 180 articles reviewed by Dijke *et al.*, 13 identified concerns over bioscientists “playing God” as an important reason for refusing to engage in germline editing.^10^ This objection has appeared widely in both the popular press^8,11–14^ and academic journals.^3,6,15,16^

This objection to a new and rapidly growing biotechnology is difficult for the scientific community to address. Many biomedical researchers have no rational or rhetorical categories for God, or for whatever it may mean to “play” in that context. In the world of genetics where scientists work long hours and rely heavily on government agencies and academic institutions for funding, such a characterization may sound ludicrous. However, one thing the scientific community cannot afford to do is to ignore the concern.

Hank Greely has noted that “using germline genomic modification to make babies will be, and should be, a political issue.”^12^ It will require an open discourse that addresses the concerns of the public, even if those concerns are outside of many scientists' frame of reference. In 2016, Elisabeth Hildt opined, “Only widely held and well informed public approval can legitimize researchers to go on in a sensitive field like this which has the power to affect society as a whole.”^9^

Herein, I wish to address this objection—advanced by the public and by academics—that applying CRISPR technology to the human germline is, or could lead to, “playing God.” Briefly, I will do three things. (1) Explore potential meanings of “playing God” through the use of CRISPR technology. How does one define “playing God” and what are the underlying concerns? (2) Test the validity of “playing God” as a potential objection to the current course of scientific development. Are arguments that bioscientists are “playing God” in this context logical? Are there counterarguments of equal or greater strength? (3) Explore how members of the scientific community might respond to this objection. How can scientists participate in the dialogue about “playing God” and have an impact on its ultimate outcome?

## What Does It Mean to “Play God”?

Criticism that scientists are “playing God” is not unique to utilizing CRISPR technologies for HGE. The birth of Louise Brown by *in vitro* fertilization in 1978 was attacked by many as unnatural or usurping the prerogatives of God.^17,18^ A 2019 study of public perceptions of genetically modifying dairy cattle so that they would be born without horns found that approximately half of respondents held a negative attitude toward the modification for “moral considerations.”^19^ The authors reporting the survey results exemplified the category of moral considerations with the quote, “I think that it is ‘playing God’ and is immoral and unethical.”^19^ The British watchdog organization, Human Genetics Alert, describes human germline editing as “playing God” and compares it to climate change in its potential for creating disaster.^20^ More direct objections to using CRISPR for human germline modification as “playing God” are also well established in the public discourse.^3,4,21^

Exactly what critics mean when they articulate the objection that scientists are “playing God” can be hard to nail down. It may or may not involve implications of deity *per se*. Some equate “playing God” with “unnatural.”^3,17^ The argument is that “naturally” produced children pose fewer risks, both for the child in question and for future generations, than children born as a result of germline editing. Natural (unedited) reproduction is also considered a positive force in the development of the human species. Powell and Buchanan interpret the argument as relying upon natural selection to be a “master engineer” whose results are unlikely to be improved upon through human intervention.^22^ The natural reproductive process, or God, is held in high regard, and scientists who intervene in the process are expected to produce suboptimal outcomes.

Michael Sandel famously criticized gene editing as an act of “hyperagency.”^23^ (Sandel does not use the term “playing God” in his arguments, but nonetheless his argument that it would represent a confusion of our role in creation with God's has received this moniker.^24^) Sandel's “hyperagency” appears to be pejorative. The prefix “hyper” comes from the Greek 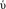
π
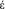
ρ, meaning “beyond” or “to excess.”^25^ Hyperagency would then mean overextended agency in the same way that hyperextending one's knee means bending it beyond the normal safe range of motion. Sandel's argument is that HGE represents agency beyond what has been consigned to humankind. Sandel identifies multiple reasons for claiming hyperagency is unethical, notably that it fails to recognize the “gifted character of human powers and achievements.”^23^

Others object to the use of CRISPR for HGE as “playing God” in a literal theological sense. A common theological doctrine of God involves dividing God's attributes into two kinds: metaphysical (or natural) and moral.^26^ God's metaphysical attributes include his omnipotence, omnipresence, and similar metaphysical qualities. God's moral attributes include his wisdom, love, justice, and other moral traits.

Those critics would argue that HGE refers to “*playing* God” because humankind may achieve some of the metaphysical attributes of God—God's ability to design children for instance. However, humankind still lacks the moral attributes of God, particularly God's infinite wisdom and love.^27^ While well-meaning parents may be trying to ensure their child a better future via germline editing, they may unintentionally infect future generations with destructive genes that might then undermine the entire human gene pool. Even worse, Persson and Savulescu argue that advanced biotechnology, such as CRISPR, could be accessed by psychopaths to cause global destruction intentionally.^28^

## An Evaluation of the Arguments

As in any process of rational exploration, it is unfair to dismiss possible arguments or theses simply because they come attached with an unfortunate moniker. Many in the science community may not have categories for “God,” but it would not behoove them to dismiss such objections merely on that basis. Here, I analyze the arguments articulated above that are associated with the phrase “playing God.”

### Human germline editing is unnatural

The argument that HGE is unnatural can be applied both for and against germline editing. Powell and Buchanan dub the argument that nature is serving in the role of God for those who would oppose genetic modification in humans as the master engineer argument or “MEA.”^22^ The argument suggests that the evolutionary process, as it has acted upon humankind, has been extremely successful. Humankind is the dominant species on the planet. The MEA also alleges that the evolutionary process is beyond humans' capacity to understand. Therefore, interfering with that process through applications of gene-editing technologies will likely result in suboptimal outcomes. Let the master engineer do its work.

Powell and Buchanan, however, enumerate a dozen deficiencies of the evolutionary process.^22^ Two significant ones are that there are many examples of suboptimal design features and that the evolutionary process favors survival not optimality. They argue that HGE has a better chance of reaching optimal results than the brute force of the evolutionary process. John Harris likewise argues that normal human reproduction produces wide variations in human genomes but also that “what human reproduction does not do very well is improve [the human genome].”^29^ The normal evolutionary process is painfully slow and unpredictable, and only reliably preferences survival.^29^

### Human germline editing will undermine life as a gift

Sandel's argument that human genetic modification will undermine our experience of life as a gift holds that HGE could produce a loss of humility, an unacceptable increase in human responsibility, and a decline in solidarity as the species begins to diverge.^23^ Sandel's arguments, however, are by their nature consequentialist arguments. He does not focus the arguments on the loss of giftedness being bad in and of itself, but rather that it could lead to the outcomes he mentions. Those potential negative consequences, however, must be weighed against the potential for positive outcomes. The potential to eliminate genetic diseases might well offset the risks of undermining our perspective on life as a gift. His argument is also subject to counterexamples demonstrating that not all gifts are good. Kass notes that “the giftedness of nature also includes smallpox and malaria.”^27^ To avoid such negative gifts, germline editing might seem a very ethical undertaking.

The argument that HGE is “playing God” because it will undercut our ability to appreciate the giftedness of life also proves too much. If HGE jeopardizes our sense of life's giftedness, why don't other forms of biotechnical agency threaten it? Vaccinations, caesarian births, and many surgical procedures might also be described as a form of human agency that diminishes the gifted nature of life. The argument that biotechnology development must be frozen at a particular point to avoid “playing God” requires an argument as to why germline editing is that point. To the extent the arguments involve a negative view of the balance of the potential risks and benefits that would suggest bioscientists should continue to research means to mitigate the risks while securing the benefits.

### Human germline editing is a categorical wrong

Not all arguments that deploying CRISPR in HGE is “playing God” are consequentialist arguments. Harris notes that “intervening in the germline of humans continues to encounter hostility that is unrelated to the expected benefit or to the safety and efficacy of such procedures.”^29^ For some critics of HGE, particularly religious critics, “playing God” is a categorical wrong, regardless of the consequences in any particular case.^30^ Attempting to exercise what they see as God's prerogatives in forming the human genome represents an ethical failure, even if the result were a child rendered free of sickle cell anemia.

This argument that HGE involves taking God's place, however, is itself theologically questionable.^31^ To view God as requiring his creative prerogatives to be protected despite the consequences requires an inferior view of God from a theological perspective. Some theologians refer to this as a “God of the Gaps” who must shrink over time as human technology and agency increase.^30^ There are robust branches of theology that would view responsible use of germline editing as a commendable act of joining God as co-creator.^30^

The very argument that germline editing is a categorical wrong may be self-defeating. The development of CRISPR technology created the possibility of HGE. Given that possibility, the ethical decision is now twofold: not only “Is it ethical to engage in human germline editing?” but also “Is it ethical to refrain from engaging in it?”^32^ If the prerogative of God in question is defined as “controlling the genetic attributes of one's offspring,” then refraining from human germline editing could be an act of playing God just as much as performing it. In either case, we would be in control of the genetic attributes of the child, whether we altered those attributes through germline editing or refrained from doing so.^33^ Since as a matter of ethics it cannot be categorically wrong both to do something and refrain from doing it, the argument may collapse upon itself.

This argument is also potentially reducible to safety and effectiveness concerns. Some in this field would go so far as to argue that we became God when CRISPR technology was developed.^32^ This assertion may be overstated, as the ability to control the human germline (even with more confidence than is currently possible) would not fully satisfy an orthodox definition of God.^34^ It does reveal, however, a potential weakness in this interpretation of the playing God argument. It is the *playing* with the powers of God that religiously motivated critics find objectionable. Humans have possessed the power of God since we discovered how to make fire (or perhaps had it stolen for us).^35^ It has always been more acceptable for human beings to use such powers in a responsible manner—compare burning a patient's clothing contaminated by the Ebola virus to burning uncontaminated clothing as a simple act of arson. It is when we use such power in the absence of the moral attributes of God (wisdom, love, etc.) that we tend to draw this particular interpretation of the criticism “playing God.” If the biotechnology community can demonstrate that it is exercising its new power of germline editing in a responsible manner—for instance, demonstrating that the intervention is both safe and effective—then it could satisfy the established criteria for the acceptability of medical interventions and avoid this criticism.

## Responding to the Arguments

Many in the science community have not, so far, been persuaded by the arguments that CRISPR permits bioscientists to “play God.” This is understandable, given that none of them proved unanswerable (see above). The first thing such scientists could do is to participate in the public debate. Multiple scientists, including CRISPR pioneer Jennifer Doudna, suggested these questions be brought forward for public scrutiny.^4^ *The CRISPR Journal* is to be commended for advancing that debate through its special edition in October 2019. Those working with CRISPR technology are uniquely placed to contribute by explaining how the technology is used and how its use can be limited without sacrificing some of its potential benefits. Such statements from the bioscience community could not only convey needed information to the public but also improve public confidence in the community.

Beyond joining the public dialogue on the arguments, using the responses above or others, bioscientists should be prepared to commit to ethical research. For critics whose concerns over “playing God” lie in consequentialist terms, evidencing the necessary discipline to avoid potential harms could do much to satisfy them. It is noteworthy that the special edition of *The CRISPR Journal* followed news of the experiments of He Jiankui, who in November 2018 described his germline editing experiments resulting in the birth of twins.^36^ Many in the CRISPR community were shocked to hear someone had conducted such a clinical application.^36^ After all, Greely had stated in 2015 that one would have to be “criminally reckless, or insane, to try to make a baby this way….”^12^ But Doudna warned that this might happen in 2017.^37^ Darryl Macer opined in 2012 that such experiments would likely be adopted in China, given its cultural acceptance of human enhancement and eugenics.^38^ Further boundary crossings of this kind would likely undermine the public perception of the responsibility of scientists in this field.

The one thing the industry should *not* do is abstain. The exuberance around potential CRISPR applications is well founded, particularly in applications around somatic genome editing. Allowing its potential benefits to be unnecessarily stalled would be like Louis Pasteur allowing the theory of spontaneous generation to be foisted on him by his critics, instead of refuting it as he did.^39^ This is a time in which science can contribute tremendously to human flourishing if, and perhaps only if, scientists can avoid the perils, and the semblance, of misusing the powers of God.
